# Challenges in Emerging Vaccines and Future Promising Candidates against SARS-CoV-2 Variants

**DOI:** 10.1155/2024/9125398

**Published:** 2024-01-25

**Authors:** Tanmay Ghildiyal, Nishant Rai, Janhvi Mishra Rawat, Maargavi Singh, Jigisha Anand, Gaurav Pant, Gaurav Kumar, Amrullah Shidiki

**Affiliations:** ^1^Department of Microbial Biotechnology, Panjab University, Chandigarh, India; ^2^Department of Biotechnology, Graphic Era Deemed to be University, Dehradun, India; ^3^Department of Instrumentation and Control Engineering, Manipal Institute of Technology, Manipal, Karnataka, India; ^4^Department of Microbiology, Graphic Era Deemed to be University, Dehradun, India; ^5^Department of Microbiology, School of Bioengineering and Biosciences, Lovely Professional University, Phagwara, India; ^6^National Medical College and Teaching Hospital, Birgunj, Nepal

## Abstract

Since the COVID-19 outbreak, the severe acute respiratory syndrome coronavirus 2 (SARS-COV-2) virus has evolved into variants with varied infectivity. Vaccines developed against COVID-19 infection have boosted immunity, but there is still uncertainty on how long the immunity from natural infection or vaccination will last. The present study attempts to outline the present level of information about the contagiousness and spread of SARS-CoV-2 variants of interest and variants of concern (VOCs). The keywords like COVID-19 vaccine types, VOCs, universal vaccines, bivalent, and other relevant terms were searched in NCBI, Science Direct, and WHO databases to review the published literature. The review provides an integrative discussion on the current state of knowledge on the type of vaccines developed against SARS-CoV-2, the safety and efficacy of COVID-19 vaccines concerning the VOCs, and prospects of novel universal, chimeric, and bivalent mRNA vaccines efficacy to fend off existing variants and other emerging coronaviruses. Genomic variation can be quite significant, as seen by the notable differences in impact, transmission rate, morbidity, and death during several human coronavirus outbreaks. Therefore, understanding the amount and characteristics of coronavirus genetic diversity in historical and contemporary strains can help researchers get an edge over upcoming variants.

## 1. Introduction

The last two centuries have witnessed incursions of three significant pandemics caused by viruses from the coronavirus family: severe acute respiratory syndrome coronavirus (SARS-CoV), Middle East Respiratory Syndrome (MERS)-CoV, and the recent SARS-CoV-2 pandemic. According to WHO fact sheets, the SARS-CoV or SARS-CoV-1 responsible for respiratory infection was first reported in Asia and resulted in an outbreak of 2002–2004, infected more than 8,098 people in 29 countries and reportedly about 9% mortality (774 deaths) worldwide [1]. Later, in 2012, a new respiratory infection, MERS, was discovered in several nations in the Middle East, Africa, and South Asia, leading to 894 causalities so far [1]. Similar to SARS, SARS-CoV-2 was discovered in 2019 and is responsible for the massive spread of the pandemic. As per WHO reports dated October 18, 2023, the viral infection has accounted for over 771 million verified cases of COVID-19, and 6.9 million deaths, and still, the struggle with the deadly virus continues [2].

Prevalence studies have shown that the number of infections and reinfections worldwide at the present rate of reported COVID-19 cases is underestimated. In many nations, testing has been curtailed, and reporting has been delayed, which contributes to this in part [2]. The deadly SARS-CoV-2 virus was isolated, and its genetic makeup was made public. As its genomic sequence became available on January 11, 2020, it prompted scientists to prepare a preventive COVID-19 vaccine as soon as possible [3–5]. The scientific community's worldwide attempts to generate vaccines have escalated since the viral nucleotide sequence became available. The international pharmaceutical manufacturers and governments collaborated further to develop SARS-CoV-2 vaccines against COVID-19 infection. The COVID-19 vaccine was developed employing technology platforms, including the use of nucleic acids, peptides, virus-like particles, recombinant proteins, live attenuated viruses, and inactivated viruses, replicating and nonreplicating viral vectors [4].

The Spike (S) protein of SARS-CoV-2 is a key protein involved in receptor recognition and cell membrane fusion process. The S protein or the whole virus were the two target antigens used in the design of the vaccines [6]. Since novel and highly aggressive strains of the SARS-CoV-2 virus have surfaced, vaccines and antibodies developed against viral infection have boosted immunity. Despite these, it is not sure whether they can work permanently against the virus and provide immunity or safeguard humans. Understanding the role of mutations in immune evasion, transmission, and virulence is as crucial as creating efficient therapies and preventive measures since the pandemic was caused by a new virus that is evolving, spreading, and changing rapidly. As a result, the current study covers variants of SARS-CoV-2, particularly variants of concern (VOCs), which will aid researchers in considering alternative strategies for preventing the spread of SARS-CoV-2 and its mutants.

The study focuses on the present level of information regarding vaccines developed against SARS-CoV-2, concerning the VOC's efficacy and safety of COVID-19 vaccinations, and prospects of alternative vaccines in protection from the existing variants and other emerging coronaviruses. We searched for keywords like COVID-19 vaccine types, VOCs, and Universal vaccines in NCBI, Science Direct, and WHO databases to review the published literature.

### 1.1. SARS-CoV-2

SARS-CoV-2, which is a causal pathogen for the fatal COVID-19 illness, has spread quickly and posed a substantial threat to public health worldwide. It belongs to the most recent significant subspecies of the Coronaviridae family and beta coronaviruses (CoV) genus within the order Nidovirales [7]. In December 2019, individuals exposed to a seafood market in Hubei province, China, were the first to be diagnosed with SARS-CoV-2. It has been suggested that the infection is probably zoonotic in origin and spreads to humans via an unidentified intermediate. A few months after the initial laboratory case was confirmed and the global escalation began, the World Health Organization (WHO) officially proclaimed the SARS-CoV-2 pandemic in March 2020 [8]. According to the International Committee on Taxonomy of Viruses (ICTV), the CoVs belong to the family Coronaviridae of the Nidovirales order. Based on genotyping and serology, the family is divided into 3 subfamilies, 6 genera, 28 subgenera, and 54 species. SARS-CoV2 belongs to the subfamily Coronavirinae [9].

**Figure 1:**
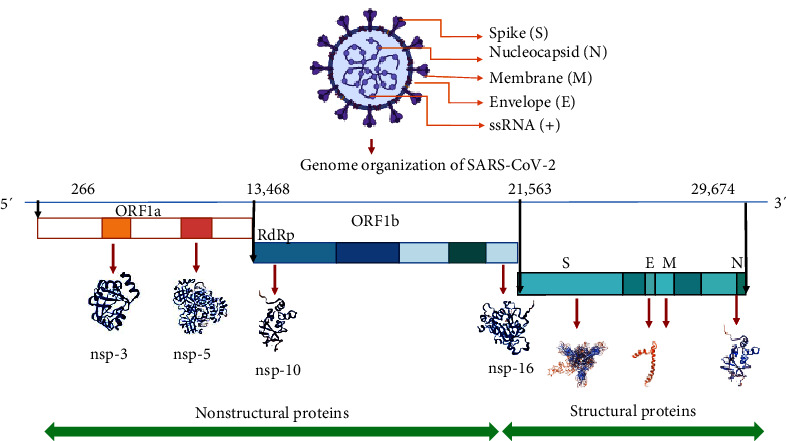
Genome organization of SARS-CoV-2.

The structure and genome organization of SARS-CoV-2 are depicted in Figure 1. The virus genome can be as large as 29.9 kb. SARS-CoV-2′s whole genomic sequence has provided the virus phylogenetic relatedness with SARS-CoV and similarity in its mode of action. According to genome-based phylogenetic analysis, SARS-CoV-2 shares 79.5% and 50% of the sequence homology with SARS-CoV and MERS-CoV, respectively [10, 11]. However, the sequence homology between the SARS-CoV-2 and other beta-CoVs is less than 9%, while seven complete replicase domains in ORF1ab of SARS-CoV-2 and SARS-CoV have only 94.6% sequence homology [11] implying the lineage B (Sarbecovirus) of beta-CoVs contains SARS-CoV-2 [12]. The 30K bp of positive-sense, single-stranded RNA genome of SARS-CoV-2 encodes for 29 proteins, including the structural proteins: nucleocapsid protein (N), an envelope protein (E), membrane glycoprotein (M), spike protein (S) and nonstructural proteins; nsp3 (ribose phosphate), nsp5 (main protease), nsp10 and nsp16 (Mtase-methyltransferase), with their probable significance in the life cycle and pathophysiology of the SARS-CoV-2 [13]. The viral proteins, like with all viruses, influence several characteristics, including attachment, egress, tissue tropism, entrance, reproduction, immunological stimulation, and continuous transmission.

### 1.2. Mutation in SARS CoV-2 and Variants Nomenclature

The CoVs were considered to solely infect animals until a SARS outbreak caused by the SARS-CoV began in China in 2002 [12]. Wuhan in China had an epidemic of a rare coronavirus (a member of the beta coronavirus cluster) at the end of 2019. During the first 50 days of the outbreak, almost 1,800 individuals died, and over 70,000 were infected. The unusual virus was given the names Wuhan coronavirus and 2019 novel coronavirus (2019-nCov) by Chinese researchers. The name SARS-CoV-2 was announced by the ICTV on February 11, 2020, and coronavirus disease for 2019 has been designated as COVID-19 by WHO following the guidelines of the World Organization for Animal Health and the Food and Agriculture Organization of the United Nations [14]. Since RNA viruses lack DNA-based systems for proofreading, they mutate more frequently than DNA viruses [15]. Many mutations were observed in the SARS-CoV-2 genome due to deletion selected point mutations, a mutation in S protein, and mutations related to the host immune system. The anticipated per-site per-year mutation rate of the human beta-coronavirus SARS-CoV-2 was estimated to be ∼1.12 × 10^−3^ nt^−1^ year^−1^ [16].

Global Initiative on Sharing Avian Influenza Data (GISAID), NEXTstrain, and the Phylogenetic Assignment of Named Global Outbreak (PANGO) sources are established systems that name and track virus variant of concern (VOC) and variant of interest (VOI) [17]. These systems provide a common language through which the scientific community can communicate, discuss, and investigate the novel SARS-CoV-2 evolution. Initially, SARS-CoV-2 variants were designated as lineages A and B, while a series of numbers labeled their descendants by PANGO. Since January 2020, linage A descendants were marked as A.1, A.2, A.3, A.4, and A.5, while in the case of B lineage first reported in December 2020, the descendants were marked as B.1, B.1.1, B2, B3, B4 [18, 19]. Later, to ease the complexity and confusion with the naming of variants among the general public or nonscientific communities, a new nomenclature was used for SARS-CoV-2 variants by WHO recommending labels using letters of Greek alphabets like alpha (*α*), beta (*β*), gamma (*Γ*), delta (*δ*), epsilon (*ε*), and omicron (*ο*) [17–19] (Table 1). On March 15, 2023, WHO declared that only VOCs would be given Greek letters, while VOIs would be referred to using recognized scientific nomenclature systems as those used by Nextstrain and Pango; for example, XBB.1.5/23A for the most recent (VOI).

### 1.3. SARS-CoV-2 Variants and Their Prevalence

The SARS-CoV-2 viral genome has undergone continual evolution, giving rise to several variants with various alterations, some of which have increased virulence compared to earlier lineages. SARS-CoV-2 variants have arisen and been discovered in numerous nations worldwide due to their global prevalence. Such viral evolution or mutations are quite concerning because they may reduce vaccine efficacy and accelerate virus propagation [20]. As a result of the SARS-CoV-2 genetic code's rapid and continual evolution and the creation of numerous variants around the world, the SARS-CoV-2 Interagency Group (SIG) focused on the quick characterization of evolving mutations. The SIG actively tracked how they would affect crucial SARS-CoV-2 countermeasures, such as vaccinations, treatments, and diagnostics. The Interagency Group is comprised of the CDC, NIH, FDA, BARDA, and Defense Department [21]. The variants have been classified into three categories: VOI, VOC, and variants being monitoring (VUM). The VOIs are the variants of SARS-CoV-2 resulting from genetic changes that effects transmissibility, pathogenesis, and effectiveness of therapeutic measures [22]. The VOCs are the VOIs of SARS-CoV-2 variants with significant public health concerns due to their associated with more severity in virulence, increase in community transmission rate, evasion of immunity and diagnosis, and reduced effectiveness of public health or social measures [22]. According to WHO, VUM describes the COVID-19 variants that undergo unusual mutagenic variations and require global tracking [22].

The PANGO lineages suggest that SARS-CoV-2 has 1542 validated lineages recorded so far [19]. The SARS-CoV-2 VOIs showed phenotypic changes, which led to variations in virulence, antigenicity, and epidemiology that could exert detrimental effects on existing diagnostic techniques, vaccinations, treatment, and social measures [23]. SARS-CoV-2 VOIs were identified with genetic markers likely to alter viral propagation, detection, treatment, transmissibility, or even the potential to partially escape immunity brought on by natural infection or vaccination [24]. Epsilon, zeta, eta, iota, theta, kappa, lambda, and mu variants were among the VOIs that had previously been in circulation [25].

Epsilon variant, or Lineage B.1.427/B.1.429, was initially detected in California, USA, in March 2020 [26]. In B.1.429, the mutation was reported in spike protein L452R, S13I, and W152C, along with the mutations in orf1ab: D1183Y and I4205V [27]. A 20% rise in transmissibility was observed. The variant's neutralization reduction was observed utilizing postvaccination and convalescent sera [28].

Zeta variant or Lineage P.2 is an alias of B.1.1.28.2 [19]. The first case of Lineage P.2 was detected in Brazil in April 2020. The mutations were detected in the spike protein L18F, T20N, P26S, F157L, E484K, D614G, S929I, and V1176F of the variant [28]. Lineage P.2 was labeled as a variant of interest in July 2021. The eta variant or Lineage B.1.525 was first detected in December 2020 in the United Kingdom and Nigeria [29]. The mutations detected in the spike protein were H69-V70 deletion, D614G, E484K, Q52R, Q677H, Y144 and, F888L deletion [28].

Theta variant, or Lineage P, was predominately detected in the Philippines in January 2021. Later, cases were confirmed in Japan and the United States. Spike mutations detected were E484K, N501Y, P681H, and 141−143del [30].

In January 2020, a new variant was detected in New York and was designated as B.1.526, or Iota variant, which later spread to other states. The characteristic mutation of the variant was spike mutations T95I and D253G [19]. Other mutations detected were L5F, S477N, E484K, D614G, and A701V [28].

The kappa variant, or Lineage B.1.617.1, is one of the three sublineages included under Lineage B.1.617. It was initially detected in December 2020 in India. The observed mutations include D614Gk E484Q, L452R and, P681R [31]. The P681R mutation has reportedly enhanced its pathogenicity because it has altered its affinity for the ACE2 receptors [24].

The lambda variant or Lineage C.37 belonged to the B.1.1.1 lineage and was first detected in Peru in August 2020. The mutations in the lambda variant included deletion (*Δ*3,675–3,677) in open reading frame 1a (ORF1a) and other deletion at *Δ*246−252. Other mutations included spike mutations D614G, F490S, G75V, L452Q, T76I, and T859N [32].

Mu variant, or Lineage B.1.621, was first reported in Columbia in January 2021. About 23 mutations were reported in the mu variant [33]. Nine of them were spike mutations namely D614G, D950N, E484K, T95I, Y144S, Y145N, R346K, N501Y, P681H, and four in ORF1a: T1055A, T1538I, T3255I, and Q3729R, two in ORF1b: P314L and P1342S, one in N: T205I, four in ORF3a: P258 ^*∗*^, N257Q, Q57H, and V256I, and three in ORF8: P38S, T11K, and S67F [34, 35].

Important pandemic factors, including enhanced transmission and decreased vaccine effectiveness, have already been significantly impacted by VOC lineages of SARS-CoV-2. The VOCs are discovered when there is evidence of higher transmissibility, more severe illness (increased hospitalizations or mortality rate), a notable decline in the number of antibodies neutralized during a prior infection or vaccination, reduced treatment or vaccine efficacy, and policies and procedures for error detection [21]. A variant with the PANGO phylogenetic nomenclature B.1.1.7 (identified as alpha according to WHO and Public Health England) was first recognized in November 2020 in the United Kingdom, which rapidly spread globally, exhibited 50% transmissibility and proved fatal to be fatal in the severely hospitalized cases [36], while another study had reported 70%–80% transmissibility in the United Kingdom between October and November 2020 [37]. A cross-sectional study in Japan indicated 1.9–2.3-fold higher dominance of the alpha variant and determined 38.7% transmissibility [38]. The alpha variant has an S protein mutation (a critical change in amino acid, Asn501 to Tyr (N501Y), within the receptor binding domain (RBD)) that enabled more accessible binding to ACE2 receptors, potentiating its infectivity. This conclusion was validated by animal models' research, which revealed that the alpha genome was more infectious to cells [39].

Additionally, S protein deletions at positions 69 and 70 resulted in the failure of virus detection in the RT-PCR diagnostic tests and, hence, doubled the infectivity and cases worldwide [40, 41]. In contrast, excision at position 144 was reported to reduce the ability of a few monoclonal antibodies to bind to the S protein antigen [42]. Furthermore, a team of researchers highlighted that the mutation in the innate immune antagonists located outside the S-protein coding region of Orf9b and Orf6 (subgenomic RNA and protein levels of N-protein) resulted in decreased host innate immune responses against the alpha variant [43].

In December 2020, another prevalent lineage in the same region, B.1.351, commonly called VOCs-20DEC-02 or beta, was first detected in South Africa before its distribution throughout the world. The rapid growth and aggressive phenotype of the beta variant were feared to compromise the efficacy of the current vaccinations [44]. The E484K alteration (or referred to as the replacement of amino acid Glutamine by amino acid Lysine at 484 positions) in the RBD domain of S protein is one of the most significant concerning mutations in beta variants, which can result in ineffective immune responses, and possibly impact the vaccine efficacy against the virus [44–47]. In a recent work published in Lancet Microbe, a team of scientists demonstrated the *in vitro* microneutralization of two almost similar viruses (the SARS-CoV-2 recombinant virus with a single E484K mutation and the USAWA1/2020 virus) [48]. According to their research, the E484K mutation can reduce the affinity of serum polyclonal neutralizing antibodies, which lowers antibody neutralization and makes it possible to evade the acquired immunity resulting from previous infections and vaccinations. In addition, the K417N mutation in the S protein also favors its ACE2 receptor binding, which boosts the variant's transmissibility [49]. Furthermore, evidence suggested that the S protein L18F mutation impairs the interaction of neutralizing antibodies, which may bring down the productiveness of vaccines and other antibody-based therapeutics [50].

In early January 2021, Brazil and Japan experienced the emergence of a novel variant, P.1 (VOCS-21JAN-02 and gamma) [51]. According to the investigations, the virus acquired numerous mutations, 12 of which were found within RBDs. These mutations included biologically significant changes to the critical residues E484K, K417N/T, and N501Y, which are also found in other variants of concern (VOCSs) [26]. The descriptive analysis of molecular characterizations of beta and gamma variants revealed that E484K and N501Y mutations enhance ACE2 receptors binding gamma S protein, resulting in increased virus infectiousness. In contrast to VOCs beta, the K417N/T transformation revealed a lower binding affinity for gamma VOCs [52].

In April 2021, another member of the VOCs group and member of the sublineage group of a related B.1.617 lineage was first identified in India, designated as B.1.617.2, VOCS-21APR-02, and delta. Noteworthy mutations, including L452R, T478K, and P681R, were linked to a potential increase in transmissibility and immune evasion of the delta variant, which made a significant global impact with an enormous spike in reported hospital-administered cases and death rate [53]. Additionally, it was further notified that by June 2021, delta was most frequently disseminated in the United Kingdom and had overtaken previously reported VOC cases, raising serious concerns about public health [54, 55]. The scientific data of research in the United Kingdom also suggested 60% more transmissibility in delta than in alpha. Additionally, the research demonstrated a 17% enhancement in the risk of COVID-19 symptoms in people following the first dose of the BNT162b2 or ChA-dOx1 vaccines in comparison to alpha; however, following the two doses of vaccine, this risk was low and observed between 4% and 6% only [56]. Based on the pattern of transmissibility and infection rate, it is crucial to stress that the B.1.617 lineage has a significant capacity to diversify into notable sublineages with various mutations [57]. The L452R and T478K mutations in all the sublineages of B.1.617 were responsible for blocking the production of antibodies induced by vaccination, as highlighted in a study led by Mlcochova et al. [58]. Similarly, the delta variation descended from the B.1.617 lineage, and various sublineages were generated from the delta variant sublineages [59].

The genome sequence discovered a novel variation known as B.1.1.7 +E484K, which was produced from the present beta variant even though other VOCSs do not appear to carry the same mutation [60]. This scenario is also a helpful illustration of currently known variants that can demonstrate how different verified variants have mutated and generated new VOCs. For instance, Collier et al. [42] reported a study in which they compared the ability of sera from individuals who had received the Pfizer-BioNTech vaccine to neutralize viruses pseudo-typed with S proteins of wild-type beta concerning controls who had beta spikes that had been altered to correct the E484K mutation (BNT162b2). These studies demonstrated that the E484K alteration led to a significant reduction in the neutralizing capacity of the vaccine by patient antibodies as well as monoclonal antibodies [42]. To confirm if VOCSs carrying this mutation are escaping, real-world data from vaccine recipients will be necessary to support in vitro results.

In November 2021, another novel variant known as B.1.1.529 (VOCs- 21 NOV-01 and omicron) appeared. This variant was instantly classified as a VOC because of its rapid dissemination and the increased number of mutations in the S protein's sequence [57, 61, 62]. B.1.1.529 variant with an alarming profile, had some changes in the genome similar to those in other VOCSs, which led to heightened immune—evasion and vital ACE2 receptors binding affinity, leading to an increase in the likelihood of contracting the disease that alerted everyone in the globe. An alarming increase in case numbers was the reason for questioning the vaccine's efficiency and the virus's rapid transmission rate. Very recently, in October 2022, its new subtype, omicron BF.7, was detected and found to possess rapid spreadibility, causing widespread illness. It could quickly bypass the immune response in individuals with natural immunity with previous variants or who have completed vaccination courses [63].

The Network for Genomic Surveillance in South Africa (NGS-SA) detected new lineages of omicron (BA.4 and BA.5) in January and February 2022, respectively, which by April 2022 had taken over as the predominant lineages in the nation. The BA.4 and BA.5 lineages, whose dominance varied geographically, caused the fifth wave of infections. Both lineages share the same spike proteins as BA.22 but differ from it by having extra mutations in the NTD (69–70 deletion) and RBD (L452R, F486V, and wild-type Q493 areas). In comparison with BA.1 and BA.22,4, these new lineages demonstrated lower serum neutralization from people previously injected with three doses of the COVID-19 vaccination (BNT162b or ChAdOx1-S). Additionally, BA.4 and BA.5 have demonstrated immunity evoked by BA.1 escape. Despite earlier waves of omicron BA.1 and BA.2, the discovery and rising incidence of BA.4 and BA.5 were reported in several nations [64]. Sublineages of omicron variant BA.5.2.1 with a mutation at amino acid R346T on the spike protein showed dominant transmissibility in July–December 2022. This novel omicron variant BF.7, also known as BA.2.75.2, has resulted in an epidemic-like situation in China and cautioned the health authorities [65]. Since then, many cases of BF.7 infection have been reported in Japan, South Korea, the United States, South Africa, Brazil, and India. Till October 5, 2022, there were 9,809 sequences of the BF.7 sublineage reported globally. As per the epidemiological study, between September 12, 2022, and October 5, 2022, there was a surge in the prevalence of BF.7 sublineages. Of these sequences, 450 of BF.7 sequences were reported from Belgium, 99 from the Netherlands, and 501 from Germany. Similarly, 319 BF.7 sequences were reported from France and 326 from the United Kingdom between September 9, 2022, and October 5, 2022 [65].

The stringent lockdown policy and vaccine hesitancy were considered reasons for driving a surge of COVID-19 infection in China. Although BF.7 has caused an outbreak in China, the variant has not resulted in an outbreak in India due to the development of hybrid immunity, herd immunity achieved through efficient vaccinations, which has helped people to fight the battle against the COVID-19 infection [66].

As of January 13, 2023, other than BF.7, other subvariants of omicron under WHO monitoring are BQ.1 (BA.5 sublineages), BA.2.7.5 (BA.2 sublineages), and XBB (Recombinant of BA.2.10.1 and BA.2.75 sublineages) [25]. According to predictions of its growth rate and genetic traits, XBB.1.5 is likely to contribute to an increase in case incidence internationally. As per the WHO database, there is moderate strength scientific evidence that indicates an increased likelihood of transmission of XBB.1.5 and its evasion to immunity from vaccination [67]. There have been no early warning signs of a deterioration in the severity, according to reports from numerous nations. Since there are only a few cases linked to XBB.1.5, the severity cannot be determined with certainty just yet. Together, the information does not imply that XBB.1.5 poses a higher danger to public health than the other omicron descendant lineages that are currently in circulation. Numerous sublineages of omicron continue to circulate, and it remains the variety that causes worry on a global scale. The omicron subvariant XBB.1.1, which has been discovered in roughly two dozen nations, is being monitored by the WHO and listed as a WHO variant under monitoring.

### 1.4. COVID-19 Vaccines

Since the outbreak of COVID-19 infection, effective therapeutics and vaccination strategies have been implemented to prevent public health worldwide. The SARS-CoV-2 S glycoproteins have a potential role in its host attachment, entry, and inducing neutralizing antibody response, therefore rendering it a prospective candidate for vaccine development. While their clinical trials are ongoing or in the planning stages, several advanced vaccination technologies, including nucleic acid, inactivated, viral vector, subunit, and recombinant vaccines, are created and studied for their therapeutic efficacy (Table 2).

#### 1.4.1. Whole Inactivated Viral Vaccine

The whole virion-inactivated SARS-CoV2 vaccine BBV152, COVAXIN, was developed by Bharat Biotech International Limited, which was granted permission to use it in an emergency to stop COVID-19. The vaccine contains 2-phenoxyethanol that preserves viral stability and contains an aluminum-based adjuvant that enhances immune response. The vaccine is administered intramuscularly in the recommended two doses. In Phase-3 trials, the vaccine had 78% efficacy after administration of the first dose, while it showed 93% effectiveness 14 days or more after the second dose. Till October 25, 2023, 169 million doses of COVAXIN were administered to the human population [62].

Sinovac-CoronaVac is an inactivated whole virion vaccine against COVID-19 developed by Sinovac Life Sciences (Beijing, China). The vaccine has been in phase 3 trials in Chile, Brazil, Indonesia, and Turkey. A large phase 3 clinical trial in Brazil showed 51% efficacy against symptomatic SARS-CoV-2 infection, while 100% efficacy was observed against severe and hospitalized individuals. Strategic Advisory Group of Experts on Immunization (SAGE), an advisory working group to WHO and member states has recommended CoronaVac safe and effective for all individuals aged above 18 and above. Furthermore, the SAGE has accepted the mixed and matched two heterologous doses of WHO emergency use listing (EUL) COVID-19 mRNA vaccine or vectored vaccine as a second dose after the use of CoronaVac as the first dose of vaccination [62].

Another COVID-19 vaccine, BIBP, also known as BBIBP-CorV, the Sinopharm COVID-19 vaccine, or BIBP vaccine, is an aluminum-hydroxide-adjuvanted, inactivated whole virus vaccine developed by Sinopharm's Beijing Institute of Biological Products. Sinopharm's WIBP COVID-19 vaccine, also known as WIBP-CorV, is also an inactivated whole virion vaccine developed by Sinopharm. As on October 25, 2023, 2.32 million people have been administered by vaccines manufactured by Sinopharm [62].

#### 1.4.2. mRNA Vaccine

These vaccines use mRNA of SARS-CoV-2 that induces cells to express SARS-CoV-2 spike proteins and stimulates immune response. The Vaccine Research Centre operated with a company called Moderna to support the viral sequence data for customization of prototype approach for SARS-CoV-2 spike protein [65]. The COVID-19 vaccine candidate named mRNA-1273 was designed and developed in early February and entered the first phase of clinical trials by March 16, 2020. On December 18, 2020, the U.S. FDA approved the NIH-Moderna vaccine for emergency use with a 94% effectiveness rate. Meanwhile, the vaccine developed by company Pfizer and BioNTech SE passed in the clinical trials. The Pfizer/BioNTech vaccine was the first vaccine approved for application in the United States against COVID-19 [66]. The Pfizer/BioNTech vaccine is the most administered comparatively. The data show that 68.5% of the world population has been vaccinated with a minimum of one dose of the COVID-19 vaccine. In contrast, 24.6% of people from economically poor countries have received at least one dose of vaccine. Globally, over 13.51 billion vaccine doses have been administered, and 2.2 million doses are administered daily [75].

#### 1.4.3. Viral Vector Vaccine

**Figure 2:**
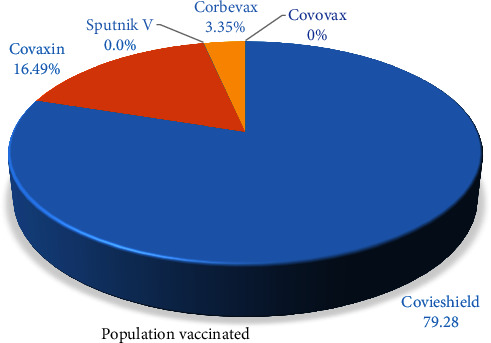
Status of vaccinations in India by several vaccine brands till October 18, 2023.

**Figure 3:**
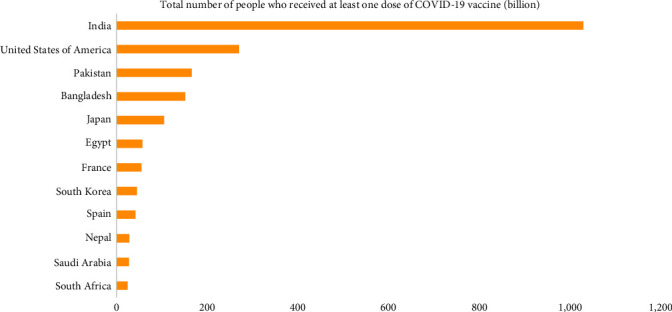
Graph depicting the worldwide status of total number of people who received at least one dose of COVID-19 vaccination till May 2023.

A viral vector vaccine, “Covishield,” is the initially approved vaccine manufactured by Oxford–AstraZeneca, produced under the license by Serum Institute of India, Pune, Maharashtra. Sputnik V (Dr Reddy's Laboratories) joined the Serum Institute of India in September 2021 to manufacture maximum vaccine jabs. Meanwhile, the clinical trials of other vaccines like the Johnson and Johnson vaccine, Moderna vaccines, and ZyCoV-D were also started. On January 16, 2021, India began the COVID-19 vaccination program phase-wise (Figure 2). According to a report published in the journal “The Lancet,” COVID-19 vaccination saved 20 million lives in a single year in India [76]. More than 2.20 billion doses of the COVID-19 vaccination have been given in India as of March 2023. The vaccination drive has been expanded to include all adults in the country, and booster doses are also being administered to certain groups. The current vaccination rate in India is around 1.5–2 million doses per day, and the government is working to accelerate the vaccination drive. Till March 2023, 87.80% of Indian citizens have received at least a single vaccine dosage, with around 94.76% of the eligible population fully immunized [42]. Currently, roughly 69.3% of the global population has been administered at least a single dosage of a COVID-19 vaccination. Globally, 13.23 billion doses have been received, with 1.17 million being distributed each day. In contrast, 6% of people in economically poor countries have received at least one vaccination dose [77] (Figure 3).

#### 1.4.4. Subunit Vaccine

Due to the high antigenicity of the S protein of SARS-CoV-2 to elicit a potent immune response, it has been considered an ideal vaccine target. A recombinant protein subunit vaccine—VAT00002 Sanofi–GSK COVID-19 vaccine has been developed by Sanofi Pasteur and GlaxoSmithKline, Belgium, using a baculovirus vector in insect cells. COVAX-19 (or SpikoGen) is another recombinant S protein vaccine candidate against COVID-19, developed by Vaxine Pty Ltd, South Africa, that has the potential to reduce infection and viral transmission [72, 73].

On November 26, 2022, vaccination against COVID-19 was observed in 218 locations worldwide [77]. Till May 17, 2022, the vaccines under EUL include the Pfizer/BioNTech Comirnaty vaccine, the SII/COVISHIELD and AstraZeneca/AZD1222 vaccines, the Moderna COVID-19 vaccine, the Janssen/Ad26.COV 2.S vaccine, the Sinovac-CoronaVac vaccine, the Sinopharm COVID-19 vaccine, the Bharat Biotech BBV152 COVAXIN vaccine, the Nuvaxovid (NVX-CoV2373) vaccine, Covovax (NVX-CoV2373) vaccine [62].  Table 3 illustrates different dosage quantities and interval recommendations for multiple vaccines.

As per the SAGE recommendation and WHO Prioritization Roadmap, EUL vaccines are safe and effective. However, they vary mainly based on the vaccine type, recommended individual age, booster dose, and efficacy against a new variant (Table 3). Till November 8, 2022, NRA (National Regulatory Authorities) has granted authorizations to forty COVID-19 vaccines with full or emergency use in more than 10 countries (Table 4). Different platforms have been used for preparing COVID-19 vaccines, including mRNA vaccines, Subunit vaccines, Inactivated virus vaccines, and Adenovirus vector vaccines (Table 4). According to Barrett's report from 2022, there have been 64 potential vaccines produced using a variety of technologies, including virus-like particles, inactivated viruses, mRNA, replication-defective viral vectors, and protein subunits. Most candidate vaccines target viral S-protein, inducing neutralizing antibodies, and have entered phase III clinical trials. A total of 175 vaccines are in clinical development, and 199 are in preclinical development [91].

### 1.5. Safety and Effectiveness of COVID-19 Vaccines

The U.S. government's SIG designated alpha, beta, gamma, and delta as variants being monitored and omicron as VOCs. Clinically severely vulnerable individuals were those thought to be most susceptible to developing severe COVID-19, including immunosuppressive conditions and severe respiratory diseases [103]. The effectiveness of vaccination is determined in a supervised clinical study by comparing the proportion of vaccine recipients who developed the disease to those who received the placebo. In the case of ChAdOx1, nCoV-19 adults who had received two doses of the vaccination showed slightly less protection against symptomatic disease with the delta variant than with the alpha variant (67.0% vs 74.5%), given over a prolonged period of 12 weeks. The ChAdOx1-S vaccine was 80.0% effective at preventing hospitalization and 84.8% against mortality following infection with the delta variant 20 weeks or longer after vaccination [68]. In the case of Ad26.CoV2. S, COVID-19 patients with symptoms that appeared at least 14 days after injection showed a 66.9% vaccination efficacy. For severe-critical COVID-19, the vaccine's effectiveness against symptoms starting at least 14 days after injection and starting at least 28 days postvaccine administration was 76.7% and 85.4%, respectively [81]. In the United States, a vaccination was 94% effective after two doses that were spaced 2 months apart. The United States had a single-dose vaccine efficacy of 72%, in contrast. Two months after immunization, the effectiveness against symptomatic illness in the single-dose study had decreased to roughly 50% [100]. After the first and second doses, Covishield's vaccination effectiveness for symptomatic patients was 58% and 64%, respectively. After more than 3 weeks of administering either dose, the effectiveness for individuals with severe symptoms was 95% [80]. Complete immunization provided a substantially more significant level of protection of 81% against moderate-to-severe disease [100].

Two doses of BBIBP-CorV administered at a 21-day interval had a 79% efficacy against SARS-CoV-2 infection that appears 14 days or more after the second treatment, according to a phase III study. In 79% of instances, immunization prevented hospitalization. There is 100% efficacy of BBIBP-CorV against severe COVID-19 symptoms [83]. According to estimates, CoronaVac is 65.9% effective at preventing COVID-19, while it showed 87.5%, 90.3%, and 86.3% effectiveness at averting hospital admission, intensive care unit admission, and COVID-19-related mortality, respectively. The results revealed that the vaccination was safe and immunogenic 14 days following the second medication for the majority of patients [98]. Two doses given at intervals of 14 days in a phase III trial in Brazil demonstrated 100% effectiveness against SARS-CoV-2 and hospitalization, while 51% against symptomatic SARS-CoV-2 infection, beginning 14 days after the second vaccination. The vaccine's efficacy against all COVID-19 variant-related illnesses for Covaxin BBV152 was 71%; its efficacy against kappa and delta was 90% and 65%, respectively. The efficacy is 81%, according to the three-stage trial [49]. The vaccine effectiveness of Covaxin in phase III clinical trials was 77.8% against symptomatic, 93.4% against severe symptomatic, 65.2% against the delta variant, and 63.6% against asymptomatic patients [85].

The mRNA-1273 vaccination revealed 94.1% effectiveness in averting COVID-19 symptoms, including severe disease [104]. After 14 days from the initial dose, the mRNA-1273 efficacy against alpha strain was 88.1% and boosted to 100% after 14 days of receiving the second dose. Following the first dose, efficiency against beta infection was 61.3%, and after the second dose, it was 96.4%. The vaccine's effectiveness after the first and second doses was 81.6% and 95.7%, respectively, against any critical or fatal COVID-19 infection caused by predominantly alpha and beta variants of SARS-CoV-2 [105]. The vaccine is still effective against viral variations. Still, the omicron variant is less effective against severe and moderate sickness after two doses than it was for delta, and its potency waned more quickly.

Adults who received two doses of the BNT162b2 vaccine demonstrated somewhat lower vaccination effectiveness against symptomatic illness with the delta variant (88.0%) concerning alpha variant (93.7%), delivered over a sustained period of 12 weeks. The effectiveness of the BNT162b2 vaccination in preventing hospitalization due to infection with the delta variant was 91.7% and 91.9% against preventing deaths [103]. Research conducted in Israel in April 2022 found that among those who had received the third dosage of the BNT162b2 vaccine at least 4 months before, the fourth dose significantly decreased the short-term risk of outcomes connected to COVID-19 [106]. After an initial vaccine series, the adoption of heterologous vaccine booster techniques lays the door for a practical, safe method of preventing catastrophic COVID-19 consequences. Flexible mRNA-based vaccination systems, in particular, have the advantage of allowing for the quick development of novel vaccines against emerging SARS-CoV-2 variants, which can subsequently be implemented right away to lower the risk of COVID-19 [107]. Despite this, several studies have reported serious adverse effects (also known as SAEs) of vaccinations based on mRNA. The findings cause worry that the potential harm from mRNA vaccines may be higher than was originally thought to be the case when authorization was granted for their global use at the time of emergency.

One Japanese study indicated that after receiving the mRNA-1273 vaccine, adverse responses were more frequently observed in females and younger people [108]. Recent case reports have revealed that non-Hodgkin lymphoma developed post the mRNA COVID-19 vaccination [109]. After receiving SARS-CoV-2 targeted mRNA vaccinations, myocarditis has been documented in children and young people in numerous nations worldwide [110]. Another study found that people with allergies had a higher frequency and intensity of adverse responses following the injection of the BNT162b2 mRNA COVID-19 vaccination. Despite this, none of the trial subjects experienced any significant adverse events, such as anaphylaxis or death. According to these findings, people who have a history of allergic reactions may still be able to tolerate the BNT162b2 mRNA COVID-19 vaccine [111]. The BNT162b2 mRNA COVID-19 vaccination was found to be safe in chronic lymphocytic leukemia patients. Still, its effectiveness is restricted in patients receiving treatment for their ailment [112]. Similarly, the BNT162b2 mRNA COVID-19 vaccine can be administered to oncologic patients safely even though they are undergoing multiple cancer treatments [113]. To satisfy the requirements of the global market, the new mRNA vaccines will have to surmount several obstacles. It is essential to continue the development of a formulation platform that can be adjusted, producing adverse effects that are even less severe or nonexistent [114].

### 1.6. Possible Reasons for the Low Effectiveness of the COVID-19 Vaccines against VOCSs

In a zero-surveillance conducted by the WHO's Unity Studies and a team of SeroTracker, 7.7% of seroprevalence was reported in June 2020, while 59.2% of seroprevalence was reported in September 2021, suggesting seropositive cases in almost 2/3rd population due to prior SARS-CoV-2 infection or vaccination [115]. The low effectiveness of COVID-19 vaccines in populations can be attributed to various reasons, of which one of many pre-COVID studies describes antigenic evolution as a key driver for positive selection for mutations in the spike protein, specifically involving RBD region, resulting in seasonal outbreaks of coronavirus [116, 117].

WHO has announced five VOCSs, which can be characterized by differing and increased spike protein mutations, building up to antigenic evolution and viral escape. Recent studies have also indicated impaired virus neutralization by polyclonal human sera due to mutations in the RBD [116]. The 1,273 amino acid long S-protein is composed of the N terminal region (S1) responsible for viral attachment and fusion. The ACE2 binding domain acts as the receptor binding unit and is considered critical in neutralizing the novel coronavirus [118]. The deletion at the N-terminal domain in antigenic regions and several of these mutations (452 and 478) may alter the immunological responses generated by receptor-binding proteins. One of the key S-glycoprotein mutations, i.e., amino acid substation D614G, has commonly occurred in all the VOCSs and has possibly been responsible for the reduced effectiveness of vaccines and increased transmissibility [5]. P681R is another key mutation at the furin cleavage site; strains carrying this particular mutation have shown increased pathogenicity in alpha and delta variants, with one manifestation being increased resistance towards neutralizing antibodies generated by immunization [5, 119].

Recipients of Ad26.COV2. S-manufactured Johnson and Johnson showed reduced effectiveness against gamma and beta strain due to a decrease in neutralizing antibody titer formed by immunization. Successively, other major vaccines also showed a similar pattern of reduction in vaccine efficiency with the evolution of different VOCs. After the second dose, the vaccine efficacy waned at 5 months; reportedly, the effectiveness of BNT162b2 was about 82.1%, 75.7%, and 75.2% for ChAdOx1 nCoV-19 at 5 months, and at 6 months, while the efficacy was 84.3% in case of mRNA-1273 vaccines at 5 months. The effectiveness of vaccines declined further among individuals aged 55 years or older and those with comorbidities. The study also showed a similar pattern of the gradual waning of vaccine effectiveness after the second shot. The effectiveness of BNT162b2 was about 82.1%, 81.6%, and 75.7% at 5, 6, and 8 months [120].

In the recipients of BNT162b2 vaccination, fluctuation in the level of IgG was observed. After receiving the initial dose, the IgG levels rise rapidly, peaking around day 60, and then gradually decline after that. In a similar vein, compared to individuals who received the BNT162b2 vaccine, those who received the ChAdOx1/BNT162b2 vaccine developed IgG antibodies at a lower level and with a faster drop. Similar patterns were seen in people who had already been harmed, indicating that prolonged time between vaccine doses causes an antibody response that is more susceptible, leading to a quick decline in IgG and IgA concentrations in human sera. As far as the B.1.1.529 omicron variant is considered, the scenario is much graver since it can escape the immune recognition system administered by the available vaccines, all of which have shown waning signs of neutralizing response over more considerable periods and gaps between immunization. This indicates that even fully vaccinated individuals with postponed dosing intervals had waning IgG levels, contributing to low T-cell response levels [121]. Since the present vaccines are being administered intramuscularly, they elicit a high IgG response, delayed and only short-lived IgA response, which has been observed in systemic circulations. This is the other probable reason mucosal immunity wanes, and the primary sterilizing immunity is not apparent since IgA is the predominant immunoglobin for respiratory mucosal surface-linked immunity, clearly indicating the reasons for the prevalence of the B.1.617.2 omicron variant.

The other probable reasons for the waning immunity coupled with increasing and accumulating spike in glycoprotein-based mutations are mainly sourced from vaccine hesitancy and vaccine access in economically low and moderate countries. These reasons contribute to the low efficiency of vaccines and ill-performing cellular immune response [122].

### 1.7. Ongoing Promising Clinical Trials for the Development of COVID-19 Vaccines

Among major vaccines developed against COVID-19, some of them have been approved in many countries for their use as emergency preexposure prophylaxis. Currently, there are around 92 vaccines in Phase 3 clinical trials, 72 in Phase II, and 66 in Phase 1 trials. These trials are being conducted in 80 countries on 242 vaccine candidates based on inactivated antigens, mRNA, protein subunit, virus-like particles (VLP), and nonreplicating vectors [123]. Currently, COVID-19 vaccines under clinical trial in some different countries include 5 in Austria, 13 in France, 14 in Cuba, 17 in Columbia, 18 vaccines under clinical trials in Argentina, 19 in Belgium, 32 in Brazil, 33 in the Russian Federation, 38 in India, 23 in Philippines, 24 in Republic of Korea, 26 in Iran, 26 South Africa, 30 in United Kingdome of Great Britan and Northern Ireland, 31 in Germany, 44 in Japan, 12 in Mexico, 15 in Netherlands, 26 in Canada, 101 in China, 111 in United Stated of America, 38 vaccines in Australia [123].

Sinovac Research and Development Co., Ltd. is currently conducting a Phase 3 clinical trial that involves prior testing of serum following primary immunization with Vero cells-based and inactivated omicron variant-based vaccine. Adults in Chile participating in a phase II clinical trial with a primary schedule of two CoronaVac® doses and two booster doses of various vaccines were previously immunized against SARS-CoV-2. A third booster dose of the omicron, trivalent, or CoronaVac® vaccination will be administered to subjects at random. The humoral immunogenicity against COVID-19 in patients who received the omicron or trivalent vaccinations will be compared with those who received CoronaVac® to determine whether the two candidate vaccines are more effective than CoronaVac® [124].

Pfizer BioNtech will assess the immunogenicity and safety of a three-dose course of BNT162b2 (B.1.1.7 + B.1.617.2) in participants without prior immunization against COVID-19. The clinical trial for the mRNA-1273-P305 vaccine assesses the immune response and safety of investigational booster vaccines that may offer protection against the COVID-19 omicron variant and other variants.

ModernaTX, Inc., under phase II/III clinical trial, is evaluating the reactivity, immunogenicity, and safety of mRNA-1273.529 and mRNA-1273.214 administered as a booster dose [125] (Table 5). China's National Vaccine and Serum Institute is running clinical trials to determine whether the recombinant COVID-19 vaccines (CHO Cell, NVSI-06-09) and the Vero cells-based inactivated vaccine have any preventative effects on people over the age of 18. Healthy volunteers over the age of 18 and recipients of two or three doses of the inactivated COVID-19 vaccine will participate in the trial [126]. A phase III observer-blind, randomized, controlled research will assess the immunogenicity and safety of a booster dose of COVOVAX (NVX-CoV2373) for COVID-19 in Indian adults who have already received the main vaccination with BBIBP-CorV [127].

### 1.8. Future Vaccine Candidates against COVID-19

The last two centuries have witnessed three Sarbecovirus outbreaks of SARS-CoV-1, SARS-CoV-2, and MERS-CoV and have challenged the scientific community with devastating health concerns. Since the outbreak of COVID-19, the SARS-COV2 virus has been developing into new variants proving to be stronger than humans. Vaccines and antibodies developed against viral infection have boosted immunity. Still, despite these, it is not certain whether they can work permanently against the virus and provide immunity or safeguard humans. The transmissibility pattern of three important VOCs—beta, delta, and omicron—was investigated in South Africa by Columbia University Mailman School of Public Health scientists. The team of scientists, Yang and Shaman, developed a comprehensive mathematical model to reconstruct SARS-CoV-2 transmission dynamics of virus variants using weekly reported COVID-19 infection cases and death rates from March 2020 till February 2022 [128]. The study reported that beta and delta variants were responsible for destroying immunity in about 65% and 25% of people, respectively, who had previously encountered the ancestral or original SARS-CoV-2 virus. In comparison, the transmissibility of beta and delta variants was 35% and 50%. The study correlates with the 27.5% reinfection rate seen during the deadly delta wave, which stole about 2.4 billion lives in India alone between April and June 2021. The highest transmissibility among VOCs was reportedly estimated in the omicron variant, which was about 95% and destroyed 56% of immunity in preinfected and vaccinated cases [128, 129].

The SARS-CoV-2 mutation from the same omicron family and B-lineage of the genome has subsequently been attributed to the emergence of a unique subvariant of omicron BF.7 in the year 2022. This subvariant has bypassed vaccine immunity and shown a rapid transmissibility rate. Experts also believe that with approaching intense cold weather, the omicron subvariant would be more infectious in the elderly and individuals with comorbidities [63]. These findings indicate that past infection, immunity, or vaccination against SARS-CoV-2 does not ensure complete safety from a reinfection caused by a novel variant, and an occurrence of another outbreak of COVID-19 infection cannot be ruled out. Except for alpha, the other three CoV types, beta, gamma, delta, and omicron, caused immunological erosion.

Furthermore, genetic variation in delta and omicron resulted in more severe reinfection despite previous exposure. The delta variant outbreak was rapidly spreading, with great transmissibility, severe sickness, and a high mortality rate. Compared with the delta variant, the transmissibility was even 100-fold greater in the case of the omicron outbreak [130]. Considering the pattern and severity of rapid infection by VOCs, predicting the occurrence, frequency, and direction of mutation in SARS-CoV-2 leads to a new COVID-19 infection is challenging. Thus, proactive planning and development of universal vaccines are required to prevent future antigenic mutations into a new variant of SARS-CoV-2 [131].

In terms of a universal vaccine, SARS-CoV-2 nucleocapsid (N-protein) could be a future vaccine candidate since the N–N-protein is a conservative, rarely mutable protein, which enables it to fight back the newly emerging variants. As a universal vaccine against COVID-19 infection, purified SARS-CoV-2 (N) protein could stimulate the immune system to produce antibodies that protect it from future infection. Researchers at the National Institute of Allergy and Infectious Diseases, Maryland, United States, are working on the potential of SARS-CoV-2 N-protein as a modulator of innate and adaptive immunity and a potential target of alternative vaccines in COVID-19 infection [132, 133]. As evident from their data, the N-protein of SARS-CoV-2 gets scattered on the surface of the host cell, which was previously considered localized within the cell itself. These N-protein transfected cells could be released and adhere to other neighboring cells by electrostatic binding and displayed significantly suitable antibody and T-cell response by sequestering chemokines and targeting Fc-expressing innate immune cells. The study strongly indicates the future vaccine candidacy of N-proteins of SARS-CoV-2 against reinfection and the occurrence of its new emerging variants.

In another breakthrough, two research teams have created chimeric vaccines that have been shown to provide excellent protection against high-risk Sarbecoviruses, including SARS-CoV-2, and VOCs like beta (SARS-CoV-2 B1.35), delta (B.1.617.2), omicron (B.1.1.529). Martinez et al. [133] treated the vulnerable aged mice with the mRNA vaccines encoded with spike proteins of multiple domains from different members of Sarbecoviruses. These multiplex-chimeric spikes elicited elevated protective neutralizing antibodies against SARS-CoV, SARS-CoV-2, beta VOCs, and Bat CoV, thus demonstrating its use as a promising future replacement vaccine for VOCs and other zoonotic coronaviruses [134]. Another study has created a unique chimeric vaccine that could support the creation of next-generation vaccinations against SARS-CoV-2 and other viruses. By creating an engineered chimeric mRNA immunogen with an extra delta variant RBD in the context of the complete S protein of omicron, the vaccine has been tested against other VOCSs like delta and omicron. The resulting chimeric mRNA vaccine induced greater protection against previous and present VOCSs, including delta and omicron, while producing noticeably larger cross-neutralization antibodies and T-cell response against delta. The chimeric architecture might make it easier to create next-generation vaccinations that balance effectiveness and coverage, not just for SARSCoV-2 variations but also for other viruses [135]. The diminished vaccine efficacy and continuous transmission of steadily emerging new SARS CoV-2 of the omicron lineage have raised the need to expand vaccine-induced immunity. The development of bivalent mRNA vaccines capable of translating spike proteins from the original and freshly surfacing variants is a promising approach towards enhancing the defense against currently circulating variants while also increasing neutralization towards the primary and potential yet-to-emerge strains [136].

Moderna's adapted bivalent vaccine mRNA 1273.211, capable of encoding the spike proteins in the Wuhan-1 and beta (B.1.351) strains, when used as a booster, was found to trigger the production of neutralizing antibodies against beta, delta (B.1.617.2), and omicron (BA.1) variants. Additionally, the antibody titers were higher than those obtained by the parental mRNA 1273 vaccination [137]. Other adapted vaccine candidates from Moderna include mRNA 1273.214, which translates the spike proteins of the Wuhan-1 variant and BA.1 subvariant of the omicron lineage (B.1.1.529). The mRNA 1273.222 vaccine encodes for spike proteins of the original Wuhan-1 and the omicron subvariants BA.4/BA.5 [138]. All these bivalent mRNA vaccines have been approved for use as boosters for individuals above 6 years of age and are being administered under Moderna's Spikevax.

Pfizer-BioNTech introduced two new adapted mRNA vaccines under the name Comirnaty, encoding the original Wuhan-1 strain and the BA.1 and BA.4/BA.5 subvariants of omicron lineage, respectively. However, the data from clinical trials of adapted Spikevax and Comirnaty vaccines (Wuhan-1 and BA.1) have been underwhelming, as the antibody titers were found to be only 1.5–1.75 times higher than those attained when the mRNA 1273 vaccine was administered as a booster [139]. Another study was conducted at Liverna Therapeutics in China, which has demonstrated delta and BA.5 subvariants S-glycoprotein encoding bivalent mRNA vaccine can generate a high titer of neutralizing antibodies against five different pseudotypes of viruses, including the original Wuhan-1 strain, and the delta and omicron subvariants [140].

Another breakthrough in the vaccine designing strategies for COVID-19 is the world's first Intranasal vaccine, iNCOVACC® (BBV154), which Bharat Biotech has developed in partnership with Washington University, St. Louis. As a heterologous booster dose, the vaccine is an adenoviral vectored vaccine with a prefusion stabilized S-glycoprotein and successfully tested in phase 1, 2, and 3 clinical trials. It is approved for a primary 2-dose schedule. Besides, Bharat Biotech is developing variant-specific vaccines to prepare against future outbreaks [141].

## 2. Conclusion

As per the available literature and scientific evidence, SARS-CoV2 will probably exist indefinitely in the environment. Therefore, the emergence of other similar zoonotic diseases from coronaviruses cannot be ruled out. Since the zoonotic virus's complete virology and geographical distribution are still unclear, rigorous studies on understanding the pathogenicity and genetic diversity of coronaviruses in multiple animals need to be accelerated. The host–pathogen relationship can be significantly impacted by mutations in any of the genes encoding viral proteins, both favorably and detrimentally. Genomic variation can be quite significant, as seen by the notable differences in impact, transmission rate, morbidity, and death during several human coronavirus outbreaks. Therefore, understanding the amount and characteristics of coronavirus genetic diversity in historical and contemporary strains can help researchers get an edge over upcoming variants.

Future risks could come from SARS-CoV-2 genetic modifications that are thought to affect the characteristics of the virus. Despite this, there is still insufficient proof of its influence on phenotypic or epidemiological consequences, demanding more observation and repeat analysis in the absence of fresh information. Considering the viral inconsistent behavior, plans for immunization are urgently needed to increase protection against the various COVID-19 strains. An essential tool for public health would be a booster vaccine that guards against COVID-19 mutations, especially the omicron form.

The COVID-19 outbreak has affected public health, resulted in fatalities, and placed a financial strain over the past 2 years. Yet, SARS-CoV-2 will likely persist in the future. Proactive planning and preparedness are paramount to mitigate its impact on a country's public health, the environment, and socioeconomic growth. The majority of the available COVID-19 vaccines have SARS-CoV-2 spike protein as their target that often mutates and modifies the SARS-CoV-2 virus into a new variant. The immunity from such vaccine candidates is less against VOCs, therefore exposing people to reinfection despite being vaccinated. Hence, alternative viral molecules are being explored for manufacturing more efficient anti-COVID-19 vaccines.

In this prospect, the development of permanent, broad-spectrum, effective antiviral universal and chimeric vaccines is necessary to evade the future biological explosion of coronavirus diseases. Moreover, rigorous investigations are required to potentiate bivalent, universal, or chimeric vaccines or as blockers of SARS-CoV-2 and its ability to prevent other deadly outbreaks. Since vaccine breakthrough cases are frequently underestimated, populations receiving all recommended vaccinations should take precautions. Continued use of preventative measures, such as masking, hand washing, isolation, and quarantine, as well as other public health mitigation tactics, genomic surveillance, and vaccination, is crucial for halting the prevalence of the COVID-19 pandemic. Early detection of variants and prevention of viral replication remain the cornerstones of the action plan.

## Figures and Tables

**Table 1:** Nomenclature of SARS-CoV-2 variants.

WHO label	Pango lineage	GISAID clade/lineage	Nextstrain clade	First documented	Types of variants
Alpha	B.1.1.7	GRY (formerly GR/501Y.V1)	20I/S:501Y.V1	UK, September 2020	VOC
Beta	B.1.351	GH/501Y.V2	20H/S:501Y.V2	South Africa, May 2020	VOC
Gamma	P.1	GR/501Y.V3	20J/S:501Y.V3	Brazil, November 2020	VOC
Delta	B.1.617.2	G/452R.V3	21A/S:478K	India, October 2020	VOC
Epsilon	B.1.427/B.1.42 9	GH/452R.V1	20C/S.452R	USA, March 2020	VOI
Zeta	P.2	GR 20B	20B/S.484K Brazil	Brazil, April 2020	VOI
Eta	B.1.525	G/484K.V3	20A/S484K	Multiple countries (UK, Denmark, Nigeria), December 2020	VOI
Theta	P.3	GR	20B/S:265C	Philippines, January 2021	VOI
lota	P.3	GR	20B/S:265C	USA, November 2020	VOI
kappa	B.1.617.1	G/452R.V3	21A/S:154K	India, October 2020	VOI
Omicron	B.1.1.529	GR/484A	21K/21L/21M/22A/22B/22C/22D	Multiple countries (South Africa, China, Hong Kong, UK, Germany, Brazil, India, etc., 2021	VOC

**Table 2:** Strategies for vaccine development against COVID-19.

Vaccine type	Technology used		Advantage	Disadvantage	Example
Viral vector [68, 69]	Harmless, modified viruses deliver specific S-protein of SARS-CoV-2 genetic material		Efficient, gene-specific delivery stimulates a more robust immune response without causing disease	Preexisting and vaccination-induced antivector immunity can interfere with vaccine immunogenicity. Immune response to viral vectors could limit its efficiency	Oxford/Astra Zeneca, Janssen/Johnson and Johnson, Sputnik Light—Gamaleya

Nucleic acid [69]	Section of genetic material (DNA/mRNA) that provides the instructions for specific proteins antigen used: S-protein		Easy and speedy designing		DNA vaccine: ZyCoV-D mRNA vaccines: Pfizer-BioNTech and Moderna COVID-19

Whole virus [62, 70, 71]	Live attenuated	Living but weakened SARS-CoV2	Stimulates strong immune response	Not safe for immunocompromised systems, the spread of CoV via the feces of vaccine recipients, risk of its recombination with wild-type CoV	Codagenix Inc., Serum Institute of India, Indian Immunological Ltd.
	Killed or inactivated	Inactivate or kill SARS-CoV2;	Easy to synthesize	Less effective, potential public health risk associated with incomplete inactivation	COVAXIN-Bharat Biotech, Coronavac-Sinovac, Sinopharm

Subunit [62, 72, 73]	Specific parts, spike proteins (the subunits) of SARS-CoV2				Novavax, Moderna

Recombinant viral vector [69, 74],	Cloned SASR-CoV-2 protein, DNA Plasmid containing SARS-CoV-2, uses recombinant S-protein mimicking SARS-CoV-2 protein as a vaccine. DNA plasmid containing SARS-CoV-2 S gene				Inovio Pharmaceuticals DNA plasmid-based prophylactic vaccine (INO-4800)

Virus-like particle-based vaccine (VLP) [69]	Multiprotein supra-molecular preparations with virus-equivalent features		(Under preclinical studies)		Medicago Inc, Saiba GmbH

**Table 3:** Schedule for different COVID-19 vaccines.

Vaccine	Dose	Interval (days)	Booster requirement
ChAdOx1 nCoV-19 [76–81]	2	56–84	A first booster after 4–6 months of primary series. Vaccine efficacy against asymptomatic and moderate SARS-CoV-2 infection gradually gets weak over time
Ad26.CoV2.S [81]	1	—	—
BBIBP-CorV [6] [82, 83]	2	21	A first booster after 4–5 months of primary series
BBV152-Covaxin [4] [84, 85]	2	28	A first booster after 4–6 months of primary series. Vaccine efficacy against asymptomatic and moderate SARS-CoV-2 infection gradually gets weak over time
CoronaVac [86]	2	14	A first booster after 4–6 months of primary series.
mRNA-1,273 [7]	2	28	A first booster for the highest priority use group (after 4–6 months of primary series). A second booster after 4–6 months of the first booster dose
BNT162b2 [87]	2	21	First booster for highest priority group (after 4–6 months of primary series). A second booster after 4–6 months of the first booster dose
Gam-COVID-Vac [88]	1	—	A first booster after 21 days of the primary dose
NVX-CoV2373 [89]	2	56 days	A first booster dose after 4–6 months of the initial series. A second booster dose is 4–6 months after the first booster dose

**Table 4:** Comparison of efficacy and safety of various COVID-19 vaccines.

Vaccine brand	Platform	Efficacy	Safety	Impact on transmissibility	Effectiveness against variants	References
Pfizer–BioNTech (mRNA-1273)	mRNA vaccines	High against severe infection, moderate against symptomatic disease	All individuals aged 6 months and above. Rare serious adverse event of mild myocarditis in young males aged 18–35 after the second dose	Modest	Low against for omicron variant	[63, 90, 91],
Moderna vaccines (BNT162b2)		High against severe infection, Moderate against symptomatic disease	Vaccine for emergency use. Rare mild myocarditis in young males aged 18–35 after the second dose	Modest	Low for the omicron variant	[63, 5]

Novavax (NVX-CoV2373)	Subunit vaccine	High against severe infection	All individuals aged 12 and above. Infrequent adverse severe events of anaphylaxis, paresthesia, hypesthesia, myocarditis, and pericarditis were reported	No substantive data are available	High against alpha, beta, and delta variants. No data are available for the omicron variant	[92, 93]

Soberana plus vaccine (Soberana 02 vaccine)	Conjugate vaccine (recombinant RBD conjugated to tetanus toxoid)	High against severe infection, moderate against symptomatic disease	All individuals aged 19–80 years, for emergency use only, transient or mild discomfort	Modest	Effective against delta variant. Data are not available for other variants	[63, 94],

Sinopharm Beijing Bio-Institute of Biological Products (BBIBP-CorV)	Whole inactivated virus	Efficacy trials are underway, but data are not available yet	All individuals aged 18 and above. Not suitable for an individual with a history of anaphylaxis	No substantive data are available	No substantive data are available	[95, 96]

Oxford/AstraZeneca ChAdOx1-S (ChAdOx1 nCoV-19/ Covishield)	Recombinant vaccine	High against severe infection and symptomatic infection	All individuals aged 18 and above. Not suitable for an individual with a history of severe allergic reactions. Rare cases of Guillain–Barré syndrome (GBS), thrombosis with thrombocytopenia syndrome (TTS) were reported	No substantive data are available	Effective against alpha variant No substantive data available for other variants	[97]

Sinovac-CoronaVac (CoronaVac)	Whole inactivated virus	High against severe infection	All individuals aged three and above. Mild and local side effects. It is recommended for emergency use only	No substantive data are available	No substantive data are available	[98, 99]

Janssen Ad26.COV2.S	Nonreplicating adenovirus vector	High against severe infection	All individuals aged 18 and above. Not suitable for an individual with a history of severe allergic reactions. Rare cases of Guillain–Barré syndrome (GBS), thrombosis with thrombocytopenia syndrome (TTS) were reported	No substantive data are available	High against B1.351 and P.2. No data available for the omicron variant	[63, 100]

Bharat Biotech BBV152 COVAXIN	Whole inactivated virus	High against severe infection, moderate against symptomatic disease	All individuals aged 18 and above. Not suitable for an individual with a history of anaphylaxis	No substantive data are available	High against all variant-related COVID-19 diseases. No data yet for omicron	[96, 101],

Sputnik V or Gam-COVID-Vac	Nonreplicating adenovirus vector (first registered combination vector vaccine)	High against severe infection, not reported against symptomatic disease	All individuals aged 18 and above	No substantive data are available	No substantive data are available	[96, 102],

**Table 5:** List of some COVID-19 vaccines under phase-3 clinical trials.

Vaccine name and developer	Country	Type	Total number of trials	Number of countries approved for use
COVAXIN-Bharat Biotech	India	Inactivated	16	14
Coronavac-Sinovac	China	Inactivated	42	56
Sputnik Light—Gamaleya	Russia	Viral vector—nonreplicating	7	26
Oxford-AstraZeneca (Vaxzevria)	United Kingdom–Sweeden	Viral vector—nonreplicating	73	149
Serum Institute of India Covishield (Oxford/ AstraZeneca formulation)	India	Viral vector—nonreplicating	6	49
Sanofi/GSK VidPrevtyn beta	United States–Germany	Protein subunit	3	30
Novavax	Australia	Protein subunit	22	40
Recombinant COVID-19 Vaccine National Vaccine and Serum Institute	China	Protein subunit	3	1
Moderna mRNA-1273.214	United States of America	RNA	5	38
Pfizer/BioNTech Comirnaty (BNT162b2)		RNA	100	149
